# Self-Reported Provider Safety in an Urban Emergency Medical System

**DOI:** 10.5811/westjem.2015.2.24124

**Published:** 2015-04-02

**Authors:** Molly Furin, Laura J. Eliseo, Breanne Langlois, William G. Fernandez, Patricia Mitchell, K. Sophia Dyer

**Affiliations:** * Boston Medical Center, Department of Emergency Medicine, Boston, Massachusetts; † Albert Einstein Medical Center, Philadelphia, Pennsylvania

## Abstract

**Introduction:**

Emergency Medical Service (EMS) personnel often respond to dangerous scenes and encounter hostile individuals without police support. No recent data describes the frequency of physical or verbal assaults or which providers have increased fear for their safety. This information may help to guide interventions to improve safety. Our objective was to describe self-reported abuse and perceptions of safety and to determine if there are differences between gender, shift, and years of experience in a busy two-tiered, third service urban EMS system.

**Methods:**

This was a secondary analysis of an anonymous, cross-sectional work safety survey of EMS providers. This survey included demographics, years of experience, history of verbal and physical assault, safety behavior following an assault and perceptions of safety. Descriptive statistics were generated.

**Results:**

Eighty-nine percent (196/221) of EMS providers completed the survey. Most were male (72%) and between the ages of 25 and 50 years (66%). The majority of providers had worked in this service for more than five years (54%), and many for more than ten years (37%). Verbal assaults were reported by 88% (172/196, 95% CI [82.4%–91.6%]). Although 80% (156/196, 95% CI [73.4%–84.6%]) reported physical assaults, only 40% (62/156, 95% CI [32.4%–47.6%]) sought medical care and 49% (76/156, 95% CI [41%–56.6%]) reported the assault to police. The proportion of those who sought medical care and reported the assault to the police was not the same across years of experience (p<0.0001). Fear for personal safety was reported by 68% (134/196, 95% CI [61.6%–74.5%]). There was no statistical difference in assault by gender; however, females feared more for their safety compared to men (38/50, 76% v 96/142, 68%, p=0.02). The proportion of those who have ever been physically assaulted was not the same across shift worked (p=0.01).

**Conclusion:**

The majority of EMS providers surveyed reported an assault and certain groups had a higher rate of assault. Most assaults were not reported to the police and medical care was infrequently sought following an event. The majority of providers reported feeling fear for their personal safety. Further research into enhancing safety mechanisms is needed.

## INTRODUCTION

In recent years, work-place safety has come into the spotlight as an important topic that needs to be addressed, especially in healthcare.^1^–^4^ While workplace violence permeates all fields of work, healthcare providers are at increased risk for violent events.^2^–^4^ Emergency medical professionals may be particularly vulnerable to such violence. In an online survey among emergency medicine (EM) residents and physicians 78% of respondents reported at least one act of workplace violence in the previous 12 months and 21% reported more than one type of violent act.^5^ While the most common type of violence was verbal threats (75%), physical assaults represented 21% of violent acts. Unlike other EM providers, much of an emergency medical services (EMS) provider’s work occurs out of the hospital, in patients’ homes, public spaces and on the streets. In the hospital, greater public safety measures have been established in many areas, including increased security officers, less after-hours access to facilities, improved surveillance, employee safety training, and in some hospitals even metal detectors at key entrances.^3^,^6^ Additionally, methods which may be employed to subdue hostile or aggressive patients in the emergency department (ED) or inpatient hospital settings are essentially unavailable to paramedics and emergency medical technicians (EMTs). In the ED, patients may be physically restrained by multiple security officers if deemed necessary; however, EMS providers are often outnumbered by patients and bystanders on the scene. The need for police back-up may not be apparent during the initial call-taking leading to a delay in the arrival of these services. EMS providers may also be unable to chemically restrain patients with sedative agents, which physicians administer if necessary for patient or provider safety. The fact that hostile or out-of-control patients may have a significant underlying medical illness that is contributing to their behavior, such as hypoglycemia, metabolic disorders, infections, or head injuries also complicates the issue.

The public calls upon EMS providers to respond to a variety of emergency situations in many different environments. Although dispatchers attempt to supply the responders with an accurate account of the incident, information relayed by patients, families, and other parties is often insufficient or inaccurate. Attempts are made to dispatch law enforcement officers or other back-up services if appropriate, but often times the two ambulance providers may be the only emergency services at a scene.^7^ In addition, emergency calls that do not initially appear to involve violence may escalate with patients, family members, or bystanders becoming aggressive or hostile. Other than training in management of aggressive behavior and scene safety, EMS providers may have few other tools to protect themselves or their patients.

Violence toward EMS providers was recognized in 1993 when Tintinalli published the results of a survey distributed to registrants of the National Association of EMS Physicians (NAEMSP) national conference.^8^ That study demonstrated that while many prehospital providers reported injuries due to violent patients, few systems had protocols for managing violent patients or formal training for recognizing and responding to violent encounters.^8^ Two years later 90% of EMS personnel in a fire-based system reported a history of violence directed toward them while at work, and abuse and violence was ranked as the top job stressor.^9^ In 1998 Corbett and Grange published that 61% of EMS providers in a Southern California system reported assaults while at work, with 25% reporting injuries from the assault.^10^ In the same system, Grange and Corbett reported violence aimed at prehospital care providers in 4.5% of patient encounters.^11^ An urban fire-based EMS system reviewed all injuries reported over a two-year period in 2002 and found that only 4% were the result of assaults.^12^ However, this study by design did not include physical assaults that did not result in injuries or were not formally reported or any verbal assaults.

Studies of international ambulance services reveal similar results. A survey of prehospital providers in Paris, France, found that 88% of respondents had been victims of a verbal threat and 41% a physical threat, yet only 9% reported formal training in managing violence.^13^ Eighty-three percent of Swedish paramedics surveyed responded that they were threatened or subject to violence, and 67% stated that they were subject to physical violence.^14^ A recent survey performed in Australia reports 87.5% of paramedics responding had experienced at least one form of violence associated with the work place in the past year.^15^

With the known risks involved in providing prehospital emergency care, changes such as improved training in personal safety and management of aggressive behavior, as well as systems for reporting violence and abuse, may have improved EMTs’ and paramedics’ perceptions of safety and exposure to violence in the U.S. This study attempts to quantify self-reported abuse among paramedics and EMTs in an urban EMS system, safety behaviors following assaults, and perceptions of safety among EMS providers. It also describes differences in reports of abuse and perceptions of safety among different groups of providers, such as gender, years of experience with the service, and shift worked. Knowledge of the frequency of assaults and factors associated with perceptions of safety may help to guide interventions to improve provider safety.

## METHODS

### Study Design

This study was a cross-sectional, anonymous survey on various safety measures among EMS personnel (EMTs and paramedics) in a two-tiered, urban EMS system. The portion of the survey reported here includes history of physical and verbal assaults, as well as perceptions of safety in the prehospital setting. A convenience sampling of participants completed surveys during required EMS clinical education sessions. This study was deemed exempt from the local institutional review board.

### Characteristics of the Sample Population

The survey was distributed to field-level providers of a two-tiered, urban EMS system in New England. This is a third service system that responds to greater than 100,000 responses per year, making it a busy, urban environment.

### Eligibility: Inclusion and Exclusion Criteria

All full-time active field providers who attended the required training sessions were asked to participate. These providers are Advanced Life Support (ALS) and Basic Life Support (BLS) providers who respond in a transport vehicle. Each employee was allowed to complete only one survey. Non-clinical providers, such as managers and administrators, and new employees (<90 days) were not included in the study population.

### Procedure

We conducted this study over a three-month period of time. This anonymous, self-administered survey was distributed during required training sessions held twice weekly during each of the three shifts. The intent was to allow participation by as many and as varied a group of providers as possible.

### Survey Instrument

We designed the survey to assess self-reported abuse and perceptions of safety in prehospital providers. Analysis of other sections has been published previously.^16^ The survey included the following sections: demographics (age, gender, and professional designation), years of experience, shift worked, history of verbal and physical assaults, incidents reported to police, incidents in which medical care was obtained, and perceptions of fear for personal safety. We measured providers’ perceptions of fear for personal safety using a Likert scale. The survey was previously tested among a group of senior EMS leadership and EMS emergency physicians. The primary outcome was the occurrence of verbal and physical assaults.

### Data Analysis

We generated descriptive, univariate statistics for all demographic variables and for the primary outcomes to determine proportion of self-reported physical and verbal abuse. We used chi-square test (Fisher’s exact when appropriate) to compare history of injury and perceptions of safety to gender, shift worked, and years of experience. Statistical significance was determined at the α=0.05 level. We conducted all analyses using SAS 9.3 (SAS Institute, Cary NC).

## RESULTS

A total of 196/220 (89%) EMS providers completed the survey. Of those respondents, 142/196 were male (72%): 37% (72/196) reported working the day shift, 30% (59/196) the evening shift, and 23% (46/196) the night shift. Most providers were between the ages of 25 and 50 years (129/196; 66%). The majority of providers had worked in this service for more than five years (105/196; 54%), and many for more than 10 years (72/196; 37%). The time with the service ranged from one to 38 years. Of all respondents, 68% (134/196, 95% CI [61.6%–71.5%]) reported that they had feared for their safety while at work (Table). Eighty-eight percent reported that they had been verbally abused or threatened (172/196, 95% CI [82.4%–91.6%]), and 80% reported that they had been physically assaulted while at work (156/196, 95% CI [73.4%–84.6%]). Overall, 40% reported that they went to the hospital post-physical assault (62/156, 95% CI [32.4%–47.6%]), and 49% (76/156, 95% CI [41%–56.6%]) replied that they reported the assault to the police.

**Figure 1:**
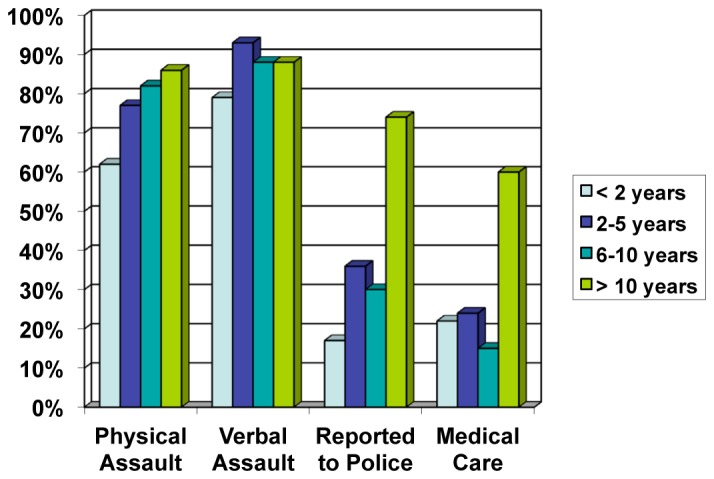
Percentage of assaults and safety behaviors by years of experience.

When separated based on years of experience, providers with two or more years of experience were more likely to have been victims of physical assault (Figure 1). Eighty-six percent (62/72) of providers with greater than 10 years experience reported a history of physical assault, compared to 82% (27/33) with 6–10 years of experience, 77% (33/43) with 2–5 years of experience, and only 62% (18/29) with less than two years of experience (p=0.03).

Only 21% of providers with less than 11 years of experience sought medical care (16/78) post-assault compared to 60% of providers with 11 or greater years of experience (37/62) (p<0.0001). (Table). In addition, only 29% of providers with less than 11 years of experience reported assaults to the police (23/78) as opposed to 74% of providers with 11 or greater years of experience (46/62) (p<0.0001). Providers with greater number of years of experience were also more likely to have feared for their safety while at work; 69% (50/72) for greater than 10 years experience, 82% (27/33) for 6–10 years experience, 67% (29/43) for 2–5 years of experience, and 52% (15/29) for providers with less than two years of experience (p=0.05).

When comparing responses by gender, 76% of females reported having feared for their safety at work (38/50) compared to 68% of males (96/142) (p=0.025) (Table). However, there was no statistical difference in reported rates of verbal abuse or physical assault by gender. There was also no difference between males and females in terms of seeking medical care at the hospital post-assault or in reporting assaults to the police.

**Figure 2:**
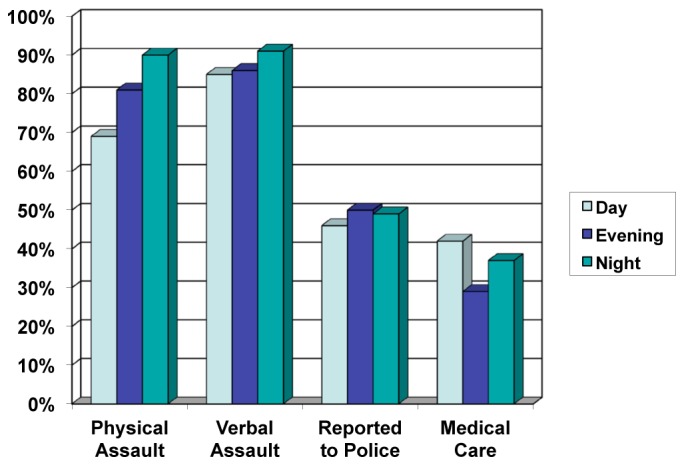
Percentage of assaults and safety behaviors by shift worked.

Responses were also compared by shift worked, and showed that fewer assaults occurred during day shift compared to both evening and night shifts (Figure 2). Only 69% (50/72) of day-shift workers reported an assault compared to 81% of evening-shift workers (48/59) and 89% of night-shift workers (41/46) (p=0.013). Rates of reporting assaults or seeking medical care were not significantly different based on shift worked.

Providers were asked to rate how safe they felt at work compared to one year prior. Sixty percent reported feeling equally safe compared to the year prior; 14% reported feeling “somewhat unsafe;” 4% reported feeling “not very safe at all;” 8% replied that they feel “somewhat safer;” and only 4% reported feeling “much safer.”

## DISCUSSION

Violence toward prehospital providers has been described previously but recent data on the prevalence of assaults and safety behaviors is lacking.^10^–^15^ This study found that more than two-thirds of professionals in EMS in an urban system have feared for their safety while at work, and that upwards of three-quarters of providers have been assaulted. Unfortunately, with such high frequency of violence, providers may have come to view threats and violence as “part of the job.” Providers may not report assaults to authorities or seek medical care unless the safety environment of each organization stresses a policy of not tolerating acts of abuse. EMS workers are responsible for delivering quality medical care to an entire community, and personal safety should be a high priority. Based on this survey, rates of assault toward EMS providers remain unacceptably high.

The data from this survey demonstrate that certain groups of employees within the EMS system have a real or perceived increased risk to their safety. Evening and night workers experience increased assaults compared to day shift workers. This finding is consistent with data from another study which showed that the hours of midnight to 6:00 AM were associated with an increase in assaults on EMS providers.^17^ Female employees fear more for their personal safety than males. While this survey found no statistically significant difference in the rates of assault based on gender, previous data has shown that of the EMS providers who died by homicide, the majority were female.^18^ In addition, employees with less than 10 years of experience may be less likely to report assaults or seek appropriate medical care following an assault. These groups may benefit from the implementation of additional safety measures. While no evidence currently exists as to the best interventions to mitigate the risks of assault in this setting, additional back-up support services, alterations in dispatch procedures, different or more extensive safety gear or training, and improved reporting systems and follow up after violent calls should be explored.

In addition to physical injuries or psychological stress sustained during an assault, an increased sense of fear of assault among EMS providers may have further consequences. Providers may change their attitudes toward patients and families or may be more hesitant to intervene in certain circumstances. Patient care may be affected if providers become impaired by their lack of sense of personal safety.^19^ Over time EMTs and paramedics may experience decreased job satisfaction, which may shorten their careers in pre-hospital medicine. Further studies would be needed to evaluate the long-term impact of assaults toward this group of medical professionals.

## LIMITATIONS

Overall, the survey had an excellent response rate. However, there are other limitations to the study. Firstly, the data collection all depended on providers’ recollection of past events, which may lead to a bias either in terms of forgetting assaults that occurred or exaggerating the events that transpired. Secondly, the data were self-reported responses, which is susceptible to over- or under-reporting based on the perceived social desirability of the answers. It was not possible to corroborate data with police or hospital records, or with EMS patient care reports. Thirdly, the survey relied on respondents’ subjective perceptions of assault and safety, which may vary greatly among providers. No standard definition for physical or verbal assault was suggested in the survey. While the lack of a standard definition does introduce a possible limitation, it remains important that each provider defined assault according to his or her own sense of personal safety. It is important to identify how providers perceived the encounter as opposed to evaluating events that met a standard definition. Further studies that gather data in real time, after each ambulance call, may help to eliminate some of these limitations. Lastly, this study gathered data from one full-time paid, urban EMS organization, which dedicates training to management of aggressive behavior. Significant variability exists in EMS organizations and results may not necessarily be generalizable to other services.

## CONCLUSION

EMS providers have made a decision to dedicate their time in the service of their community. The personal safety of these emergency providers should be a high priority. This study found that a substantial proportion of providers had feared for their safety at work, with a high prevalence of verbal assaults and physical abuse being reported. Although training in managing aggressive behavior is presented, most providers do not report feeling an increased sense of personal safety. There are certain groups of providers who have an increased real or perceived risk of violence, namely evening and night shift workers and female providers. Further strategies aimed at reducing the risk of violent events may be needed to increase feelings of safety among providers, and specific groups may need to be targeted for additional risk prevention. Additional resources should be allocated to decrease the risk of violence toward pre-hospital providers and potential consequences of these violent acts.

## Figures and Tables

**Table:** Emergency medical services provider responses to survey on work environment safety by provider characteristics.

Respondent characteristics	History of physical assault	History of verbal assault	Assault reported to the police	Hospital visit after assault	Report fearing for safety while at work
All respondents (n=196)	156	172	76	62	134
Gender: male (n=142)	116	127	57	45	96
Gender: female (n=50)	38	43	19	16	38
Years at service: <2 (n=29)	18	23	3	4	15
Years at service: 2–5 (n=43)	33	40	12	8	29
Years at service: 6–10 (n=33)	27	29	8	4	27
Years at service: >10 (n=72)	62	63	46	37	50
Shift worked: day (n=72)	50	61	23	21	48
Shift worked: evening (n=59)	48	51	24	14	37
Shift Worked: night (n=46)	41	42	20	15	37
